# A Victim-Based Framework for Telecom Fraud Analysis: A Bayesian Network Model

**DOI:** 10.1155/2022/7937355

**Published:** 2022-08-31

**Authors:** Peifeng Ni, Wei Yu

**Affiliations:** ^1^School of Economics and Management, University of Chinese Academy of Sciences, Beijing 100190, China; ^2^RichAI Technology Inc., Beijing 100013, China

## Abstract

The increasingly rampant telecom network fraud crime will cause serious harm to people's property safety. The way to reduce telecom fraud has shifted from passive combat to active prevention. This paper proposes a victim analysis and prediction method based on Bayesian network (BN), which models victims from age, gender, occupation, marriage, knowledge level, etc. We describe the fraud process in terms of whether to report to the police, property loss, and realizing the reasoning of the whole process of telecom fraud. This paper uses expert experience to obtain a Bayesian network structure. 533 real telecom fraud cases are used to learn Bayesian network parameters. The model is capable of quantifying uncertainty and dealing with nonlinear complex relationships among multiple factors, analyzing the factors most sensitive to property damage. According to the characteristics of victims, we conduct situational reasoning in the Bayesian network to evaluate property damage and alarm situations in different scenarios and provide decision support for police and community prevention and control. The experimental results show that male staff in government agencies are the most vulnerable to shopping fraud and women in schools are the most vulnerable to phishing and virus fraud and have the greatest property loss after being deceived; victim characteristics have very limited influence on whether to report to the police.

## 1. Introduction

Since 2013, criminal cases related to Internet fraud in China Telecom have been increasing, causing huge economic losses and threatening social stability. Statistics from the Ministry of Public Security show that the means of telecom fraud in China are constantly changing [1], and the number of telecom frauds is increasing at an annual rate of 20% to 30%. From 2011 to 2015, the number of telecom fraud cases nationwide surged from 100,000 to 600,000. Public economic losses soared from more than 4 billion Yuan to 22.2 billion Yuan. At the same time, the consequences of telecommunications fraud have also become a focus of social concern. For example, a sophomore student in Shandong Province died of cardiac arrest due to selfblame after being deceived.

**Figure 1:**
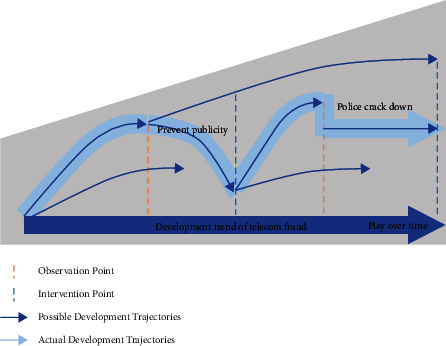
Development trend of telecom fraud.

The whole process of telecom fraud is shown in Figure 1, and the fraud trend will change in different years and seasons. At some specific point in time (vertical dashed line), due to internal and external factors, there is a certain probability that large-scale telecom fraud will occur in many possible directions (dark blue arrows). But because of the establishment of police intervention points (orange vertical lines), the actual path of telecom fraud (indicated by light blue shaded arrows) will change. The public security department can strategically intervene at key time points (indicated by orange dotted lines) to change the development trend of telecom fraud, so as to avoid the occurrence of telecom fraud or minimize the threat. For example, at the first intervention point, large-scale antifraud publicity and education led to a slow decline in telecom fraud; but at the second intervention point, the police conducted targeted crackdowns, and the trend declined immediately. It can be seen that different intervention methods will lead to different development trends of telecom fraud.

**Figure 2:**
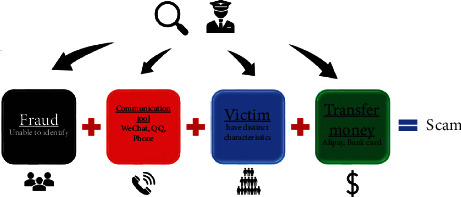
The formula for the implementation of telecom fraud.

The specific telecom fraud process is shown in Figure 2. Fraudsters of various types of fraud contact the victims in many ways, and a few victims with characteristics are successfully deceived and pay “rewards” to the fraudsters. After our research, we found that telecom fraud has three characteristics: (1) Contactless. Instead of contacting the victims themselves, the scammers communicate through some medium. (2) There are various methods of fraud. There are hundreds of fraud methods, and they are still developing. (3) Fraud is targeted. When the victim's personal information is obtained, the scammer may devise a set of special scam tricks to deceive. Due to these diverse characteristics, the combating and prevention of telecom fraud is challenging. As shown in Figure 2, the public security department can play a key role in this process: the police can cut off the source, but the crooks are highly covert. The police can filter and detect scammers, but this involves personal privacy, and the amount of data is often too large, and there is always a fish that slips through the net. Communities and police can communicate, educate and warn, which is low-cost, universal, and easy to implement. The police usually have basic information on community members and can provide timely warnings. In the process of transfer and remittance after scammers, the police can cooperate with banks and other institutions to freeze the account, which is also a common method. However, nowadays scammers often make multiple transfers, involving a small amount of money per account, and after two, three, or four transfers, the money in one account may be spread among thousands of accounts, freezing a number of accounts at one time. Thousands of bank accounts are less viable approaches. At the same time, scammers will hire professional “drivers” to withdraw and split the money from ATMs everywhere, very fast. Therefore, from the victim's perspective, an early warning can be a very effective method.

In recent years, the field of telecom fraud has become a research hotspot. In earlier research abroad, Rosset et al. proposed a fraud detection method based on rules such as suspicious behavior. They combine attributes of customers with attributes of specific behaviors that indicate fraud, use a white-box approach to generate rules, and help analysts dig out the causes of deception and design preventive measures [2]. Moreau et al. proposed a fraud prevention system based on a supervised neural network, which combines monitors and monitor thresholds, and has a prominent effect on fraud detection in mobile communications [3]. Hellas When fraudulent telecom activity occurs on the premises of large organizations, they solve the problem of detecting fraudulent behavior by building an expert system that combines the expertise of network administrators with the knowledge gained by applying data mining techniques to real data [4]. Boukerche et al. proposed a neural network classifier-based security system to detect imitators and inappropriate operations of online mobile phones [5]. However, foreign research mainly focuses on the prevention of telecommunications fraud from the perspective of telecommunications operators. At the moment when the police are the executors for prevention, the previous research lacks overall consideration. In China, the most common antifraud method is to use mobile apps such as 360 Mobile Guard and Xiaomi Mobile Manager to intelligently intercept fraudulent calls and text messages. This method is the most efficient, but because it relies heavily on fraudulent calls from security companies The quality and quantity of the number database cannot effectively deal with the frequent change of numbers by fraudsters [6]. Communication operators and other companies used template matching methods to learn from foreign experience in the early days to detect fraudulent texts and voices, but only limited to Identifying fraudulent texts based on automatic robots and the same language cannot be effectively identified based on the artificial language adjustment of fraudsters [7]. Wang and Cai proposed a data structure based on an improved GA-SVM recognition algorithm to increase the recognition effect so that telecom operators can detect in time and take appropriate measures to reduce fraud risks [8]. Xiao and Wang using Marko identified fraudulent accounts applied in telecom fraud by using the Internet of Things and extracted the RFM features of account transactions in real data to accurately identify fraudulent accounts and deal with them in a timely manner [9]. Liu and Wang proposed a dense subgraph method, which used unsupervised learning methods to detect telecom fraud [10]. The characteristics and limitations of some of the research work on telecommunication fraud prevention are shown in Table 1.

Most of the existing research methods are data-led fraud prediction and provide rich prevention methods and ideas based on the prediction results, but most of these studies use the information on fraud cases, such as fraudulent text messages, suspicious phone numbers, and fraudulent corpora. The characteristics of deceived persons in telecom fraud cases have not been quantitatively studied. In most cases, after the occurrence of telecom fraud, the corpus is unknown, and the only information that can be obtained in time is the victim's characteristics, fraudulent methods, and loss amount. In response to this problem, this paper proposes a Bayesian network model constructed from the victim's perspective, using the seven characteristics of the victim to construct a Bayesian network model for telecom fraud, which can integrate the characteristics of the victim (such as education, occupation, and age) and causal knowledge of the consequences of fraud (property damage, whether to call the police, etc.). (1) Answering questions such as “what if-how,” (2) Paying attention to the influence of important factors, making micropredictions on the suspects of electrical fraud, and evaluating the possibility of victims being defrauded and the amount defrauded.

In summary, the research contents of this paper mainly include:

1. In order to assess the risk of telecom fraud, this paper proposes a Bayesian network model for telecom fraud from the perspective of victims.
2. Based on expert experience and existing 533 typical case data, Bayesian network structure and parameter learning are carried out.
3. Four common scenarios are designed to verify the feasibility and practicability of the network model.
4. Sensitivity analysis results show that gender is male, occupation is a government agency, most vulnerable to shopping fraud; gender is female, occupation is school, most vulnerable to phishing and virus fraud; gender is female, occupation is school, property loss is the largest, gender is female, occupation is a government agency, property loss is the least; the impact of victim characteristics on whether to report to the police is very limited

The rest of the paper is structured as follows: the motivation for model selection and a brief discussion of BN is presented in Section 2. The resulting BN-based model will be described step-by-step in Section 3. Finally, the conclusions, limitations, and scope of further research of the study are discussed.

## 2. Methods

### 2.1. Model Selection

In the process of telecom fraud, the relationship between outcomes such as fraud amount and fraud type, and other process factors is often nonlinear and mixed with complex interactions [11]. For example, in Pick-up Artist (PUA) fraud cases, some victims completely trust the fraudsters because of their wealth but lack of security. After being deceived, they repeatedly believe in the other party, which leads to huge losses; can report to the police in time to reduce losses after being deceived for the first time. Data content from multiple sources is also required in order to explore the interactions and develop an effective fraudulent-oriented, fine-grained framework based on the fraud process. Such as the basic characteristics of the deceived person, the results of the deception, police data, and other subjective and objective data. Hence, data integration plays a vital role in this analysis. However, due to possible uncertainties such as incomplete data and insufficient data, the participation and judgment of experts are needed to effectively interpret and clarify the data. At the same time, model uncertainty must be considered in order to assess the consequences of fraud for a particular victim. Considering that the uncertainty of the model is also crucial for evaluating the consequences of telecom fraud, one can choose support vector machines (SVMs), decision trees (DT), Bayesian networks (BNs), and analytical network processes (ANP) based on Models of different networks to handle the relationship between parameters and account for model uncertainty.

Because SVM has the advantages of a wide application range and easy expansion, it has become the first choice for many data processing technologies in the analysis process. After the historical data is marked and labeled, the support vector machine model can be used to learn the distribution and probability of the resulting feature from the data in a supervised learning method [12], but this cannot realize the probability distribution inference between nodes; Other techniques (such as decision tree) can obtain the probability that the expected value is greater than or equal to 0 when the probability of occurrence is known in various situations, and realize the evaluation of risk consequences. Labeled data can learn certain decision logic, but there are influence relationships between different modeling factors, which cannot effectively integrate expert knowledge and historical data to achieve qualitative and quantitative unity. Because the standard of all parameters of the ANP method must be pairwise compared with other parameters, it is difficult to generate a supermatrix even with expert experience, which is not suitable for this network construction [13]. Therefore, in comparison, the Bayesian belief network can use a conditional probability table (CPT) to effectively deal with the causal relationship between key factors, and it is most suitable to build a victim network based on telecom fraud.

### 2.2. Bayesian Network (BN)

Bayesian network, also known as a belief network, is a probabilistic graphical model that represents a set of random variables and their conditional correlations through a directed acyclic graph, which is an extension of the method based on probability theory [14]. The nodes in the directed acyclic graph of the Bayesian network represent variables, and the arcs between the nodes represent the dependencies or causal relationships between variables [15]. There are two kinds of nodes in the Bayesian network, one is only a parent node and no child node leaf node, and the other is a child node, but no parent node and the root node. The Bayesian network components include Bayesian network nodes, directed links between nodes, the prior probability of each parent node, and the conditional probability of child nodes. When there are *n* random variables *m*_1_, *m*_2_,…, *m*_*n*_ and a directed acyclic graph with *n* numbered nodes, it is assumed that the node *j*(1 ≤ *j* ≤ *n*) and the *m*_*j*_ variable in the graph are related to each other. The joint probability distribution of this group of random variables *m*_1_, *m*_2_,…, *m*_*n*_ is

(1) $$\begin{array}{c} p\left(m_{1},m_{2},\ldots ,m_{n}\right)=\prod_{j=1}^{n}p\left(m_{j}|\text{parent}\left(m_{j}\right)\right), \end{array}$$

where parent (*m_j_*) represents the set of all variables *m_i_*. Based on formula (1), the conditional probability of each node in the Bayesian network can also be calculated. There are three types of methods for constructing Bayesian networks at this stage: (1) methods based on historical data learning, which calculate the scale and parameters of the network. (2) methods based on expert experience, where the network is obtained from the experience of domain experts. (3) A hybrid method based on historical data and expert experience, both of which complement each other and improve network parameters and scale [16]. A large number of experimental research results show that the application and integration of expert experience is a necessary and reliable measure to construct the Bayesian network for nodes lacking data [17, 18].

## 3. Model Building

### 3.1. Determination of Parent and Child Nodes

Based on the analysis of the introduction, this section analyzes the key points in the deception process from the perspective of the victim and conducts an assessment of the consequences. In order to explore the components and process factors in the Bayesian network model, this section combines the characteristics of the criminal behavior of telecom fraud [19] and the psychological behavior characteristics of the victims [18] to divide the deception process into three aspects.

1. A portrait of the victim. The importance of portraits in crime prediction cannot be overstated. Especially in telecommunications fraud, each type of fraud often corresponds to a certain group of people, who have some similar behavioral characteristics and psychological thoughts, and are therefore more likely to be deceived. Based on actual cases and the experience of front-line police, the characteristics summarized in this paper include age, marital status, industry, gender, and knowledge level.
2. The fraudulent process. Telecom fraud starts with a phone call, a text message, or a website link. After the victim enters the trap without any suspicion, he is confused by the tricks of the liar. The key nodes in this process are the type of scam, knowledge of the scam, and contact information. We did not put active or passive contact into the node, because through research on most cases, we found that no matter what type of fraud, passive contact is the main method, which is also in line with the “vegetable merchant” in the fraud process. The identity characteristics of fraudsters providing personal information make fraudsters actively contact victims in a targeted manner, but it is difficult to distinguish between active and passive contact methods, so we did not include them.
3. The result of fraud. The results of telecom fraud include financial loss and whether or not to report to the police. We did not consider the method of transfer and withdrawal, because we believe that when the victim is deceived, it is often ineffective to prevent from the loss. The victim usually chooses the transfer method according to the requirements of the scammer, which cannot be predicted. In fact, victims often have a certain degree of distrust in society and Internet products after being deceived. However, this is difficult to quantify, so we did not consider it.

The states contained in each child node are shown in Table 2. In the column of knowledge level, we do not divide it by educational background, because whether or not to be deceived is related to the educational background of the deceived person, but it is more about social experience and understanding of telecom fraud. We use the knowledge level to express it here. Among the types of scams, we only list seven types. These seven are the most common and frequent types, and they are representative. They account for 95% of all scams. As for the node of understanding electronic fraud before being deceived, we obtained it based on historical data statistics. Statistics show that knowledge of electronic fraud can greatly improve the vigilance of the victim. This node is also related to the portrait of the victim.

### 3.2. BN Structure Determination

We collected data on telecom fraud in a certain location for the past five years, including 533 cases. To facilitate retrieval operations, we first store the cases in a relational database. In order to further extract the relevant victim feature attribute values in each case, we wrote an automated script for data cleaning and classification, and cleaned and processed 533 telecom fraud cases in the database, in which most of the basic features in the cases were relatively complete and only a small amount of data needs to be cleaned and extracted manually. After that, we learn the parameters of the Bayesian network through the Delphi method and EM algorithm based on expert experience.

**Figure 3:**
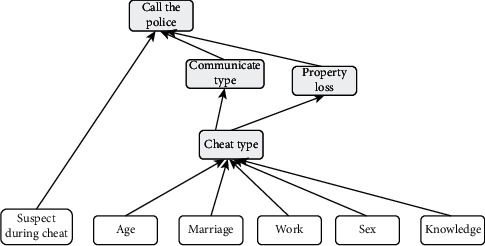
The causal relationship diagram of fraud nodes.

To leverage expert experience, we invited five experts to participate in the construction of this Bayesian network. Three of the scholars have more than five years of relevant research experience in the fields of police information, telecom fraud, and risk assessment, while two grass-roots antifraud police officers have seven years of practical experience in the front line of combating and preventing telecom fraud. First, three scholars studied and identified criteria and subcriteria suitable for telecom fraud evaluation from the literature review of the existing telecom fraud field [19, 20]. Then, two grassroots antifraud policemen were invited to verify the validity of the parent node and child node in the network. Finally, a causal relationship graph between nodes is formulated based on the experience of five experts.  Figure 3 shows the Bayesian network-based telecom fraud risk assessment model established according to the results of the expert discussions above, including 6 parent nodes and 4 independent child nodes connected by 10 causal relationships. All the variables in the figure are mutually exclusive, and finally, the specific Bayesian network construction is implemented using the GeNIe software.

### 3.3. The Probability Distribution of Nodes Is Determined

After drawing the causal graph, the prior and posterior probabilities of nodes need to be determined. A conditional probability table (CPT) is to learn the node probability distribution through expert knowledge and the MAP maximum likelihood estimation algorithm. The data is first processed in accordance with the method of machine learning, and then we ask experts to modify some probabilities that clearly do not fit the distribution to achieve the greatest practical value of Bayesian networks. In order to improve the CPT value generation process, initially, three scholars corrected the CPT value for the child nodes based on the literature review and their knowledge. Afterward, two grassroots policemen were invited to revise the CPT value based on their knowledge and experience. In overcoming the discrepancy in results, CPT values continued to vary until consensus was reached among all five experts. GeNIe considers the uniform probability of missing entries or nodes with missing or incomplete CPT.

### 3.4. Model Validation

To validate the proposed model, this paper employs a set of qualitative (scenario evolution analysis) and quantitative validation (sensitivity analysis and partial validation) methods [21]. Expert judgment plays a key role in developing and validating the constructed BN model.

#### 3.4.1. Situational Reasoning

For qualitative analysis, this section presents two scenarios for inference demonstration analysis. Among them, we use the method of BN's forward reasoning process [20] to obtain future scenarios and evaluate the future threat to the current victim. At the same time, in order to analyze the characteristics of two common groups to show the potential threat of being defrauded by different groups, we propose scenarios of two special groups, one large and one small. Adopt the method of BN's reverse reasoning process [20] to determine the characteristics of victims who are more likely to be deceived, and therefore propose corresponding antifraud publicity measures. Due to the limited space of this paper, we selected several targeted scenarios and did not consider all possible scenarios.

*(1) First Method of Reasoning: BN Forward Reasoning*. When conducting antifraud publicity, if the type of potential publicized groups can be obtained in time, then we can update the corresponding BN node probability distribution according to the BN model, and based on this, obtain the probability distribution of child nodes to achieve new reasoning. In order to target specific groups, develop preventive measures to reduce the impact of fraud. We use the following two examples to demonstrate the effect of the model:

**Scenario 1 .**

Mr. A is an operations manager of a listed technology company in city B. He is 35 years old and has two children. He graduated from University C with a major in marketing planning. One day, he came into contact with fraudulent information. A fraud case occurred within the company a few days ago.

**Scenario 2 .**

Ms. A is the operator of a tobacco store in City B. She was born in the 1970s. She went out to work after graduating from junior high school. She has no children yet. The community government will take care of her regularly. Contact, during which there is no defense against each other.

First, the corresponding content information needs to be extracted from each scenario. The status in Scenario 1 can be known as “Mr. A” = “male,” “a listed technology company” = “corporate enterprise,” “35 years old” = “middle-aged,” “there are two children in the family” = “married,” “graduated from University C” = “high educational level,” “thinking of a fraud case” = “doubtful”;

From the state in Scenario 2, we can know that “Ms. A” = “Female,” “A certain tobacco store operator” = “Selfemployed,” “Born in the 1970s” = “Old age,” “No children under the knee” = “unmarried,” “going out to work after graduating from junior high school” = “low level of education,” “no defense against each other” = “no doubt.”

**Figure 4:**
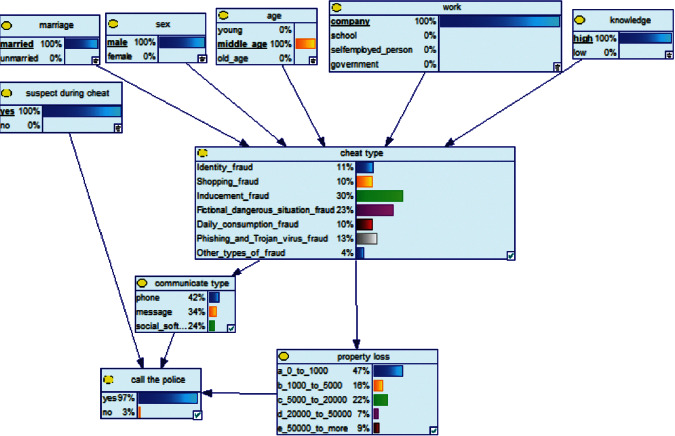
Scenario 1 Bayesian network probability distribution.

We substitute the above cases into the Bayesian network model, respectively, and derive the posterior probability of other subnodes. The distribution results are shown in Table 3. At the same time, combined with the content of Figure 4, it can be seen that the most likely type of fraud in Scenario 1 is the bait-and-seek fraud case, with a probability of 30%. This is because this type of group is greedy for petty gain. In addition, the most likely property loss is the range of the amount from 0 to 1,000 Yuan has a probability of 47%. According to common sense analysis, it can be seen that this group of people has a certain awareness of prevention, and has the behavioral characteristics of being cautious about the outflow of large sums of money, but at the same time, there are psychological behaviors of greed for petty gains and gullibility.

**Figure 5:**
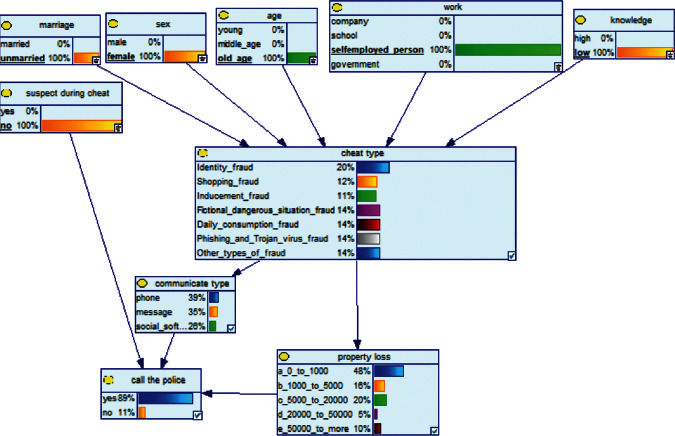
Scenario 2 Bayesian network probability distribution.

Combining with the content of Figure 5, it can be seen that the most likely type of fraud in Scenario 2 is fake identity fraud with a probability of 20%. This is because this group usually has no children and no daughters to take care of, so it is easy to trust others and to be deceived by someone else's fictitious identity. At the same time, the most likely property loss is in the range of 0 to 1,000 Yuan, and the probability is 48%. According to common sense, it conforms to the maximum “fund usage” acceptable to this group, and it also shows the harm of such fraud. The gender is not high but the coverage is wide, which is easy to reduce the trust of the elderly in the society. According to the probability distribution of the child nodes of the Bayesian network, the most common response to each type of victim is to call the police. Therefore, the measures that can be formulated include extensive publicity in advance and timely alarm after the event. In particular, targeted publicity can better reduce hazards.

At the same time, we evaluate the degree of loss in telecom fraud cases. The formula for evaluating the property loss of telecom fraud is as follows:

(2) $$\begin{array}{c} \text{Per capita loss}=L_{1}P_{m1}+L_{2}P_{m2}+L_{3}P_{m3}+L_{4}P_{m4}+L_{5}P_{m5}+L_{6}P_{m6}. \end{array}$$

As shown in Table 3, *L*_1_, *L*_2_, *L*_3_, *L*_4_, and *L*_5_ represent the state of the property loss node, and *P*_*m*1_, *P*_*m*2_, *P*_*m*3_, *P*_*m*4_, and *P*_*m*5_ represent the probability based on the state of each node in the Bayesian network. Based on this, we calculate the amount of loss.

We use the formulas above to calculate the worst, best, and average outcomes for scams. Considering that there is no upper limit of the amount in the L5 state and cannot be evaluated, we use the historical data of the maximum amount of 1 million Yuan as the upper limit for calculation. Where we take the maximum value of each state to calculate the worst result, the median value to calculate the average result, and the minimum value to calculate the best result. For example, in the L1 (0–1000 Yuan loss) state, the maximum value is 470 and the average value is 235. The best result is the median value of the state variable with the highest probability value. For example, in this state, the maximum probability state of the lost node is 47% of L1, and then we take the median of 235 Yuan as the best fraud outcome, ignoring the probability state of others. Through the above method, we get the severity of fraud cases. The consequences of the fraud calculation in this state are shown in Table 2. The property loss is 14,710 Yuan, with an average of 8,550 Yuan and a minimum of 98,970 Yuan.

*(2) Second Method of Reasoning: BN Reverse Reasoning*. In the field of electricity fraud prevention, the reverse reasoning process is used to conduct reverse mining from the threat results to find out the characteristics of the victim, and on this basis, intervene and take targeted precautions to reduce the threat of fraudulent consequences. We will assume two examples to illustrate the effectiveness of this Bayesian network model.

**Scenario 3 .**

Due to the pervasiveness of telecom fraud, the public security organs have conducted many antifraud presentations in the community and explained many cases of telecom fraud. Being cheated often happens. One day, there was a fraud in a community that faked the identity of the public prosecutor, and the victims were defrauded of more than 50,000 Yuan in succession.

**Scenario 4 .**

The main reason why telecom fraud is not finished and eliminated by the public security organs is that the fraudsters often use small amounts and widespread nets to defraud, and often catch the victims who are greedy for petty benefits and cheated. The psychological behavior of reluctance to report to the police because of the small amount. One day, there was a deception in a community of paying a deposit on the grounds of winning a lottery, and the victim paid a deposit of 700 yuan.

**Figure 6:**
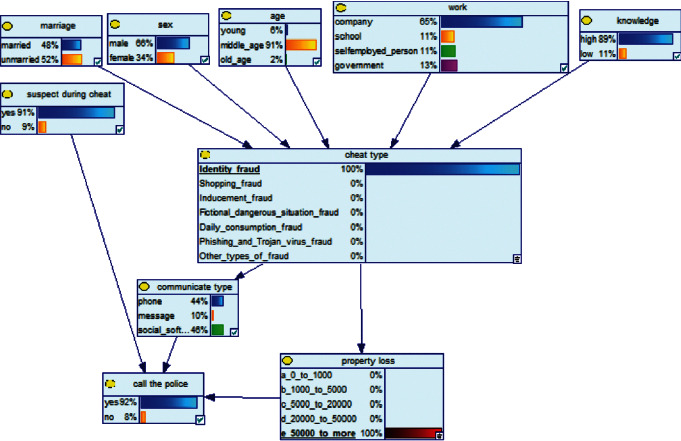
Scenario 3: Bayesian network probability distribution.

From the situation in Scenario 3, we can learn the following information: “Fraud impersonating the identity of the Public Procuratorate” = “Identity impersonation fraud,” “Amount defrauded of more than 50,000 yuan” = “Property loss of more than 50,000 yuan”; from Scenario 4 Under the condition of stacking, we can learn the following information: “pay a deposit on the grounds of winning the lottery” = “baiting fraud,” “pay a deposit of 700 yuan” = “property loss is less than 1,000 yuan.” We checked and changed the corresponding child nodes, and after updating the Bayesian network, the posterior probability of each parent node was obtained. The probability distribution is shown in Table 4. Combined with the content of Figure 6, it can be seen that under the situation of Scenario 3, the probability of the victim being male is 66%, the probability of being middle-aged is 91%, and the probability of working in a company is 65%. The probability is 89%. According to common sense, this is consistent with the basic situation of victims of this type of fraud, and they have high financial control rights.

**Figure 7:**
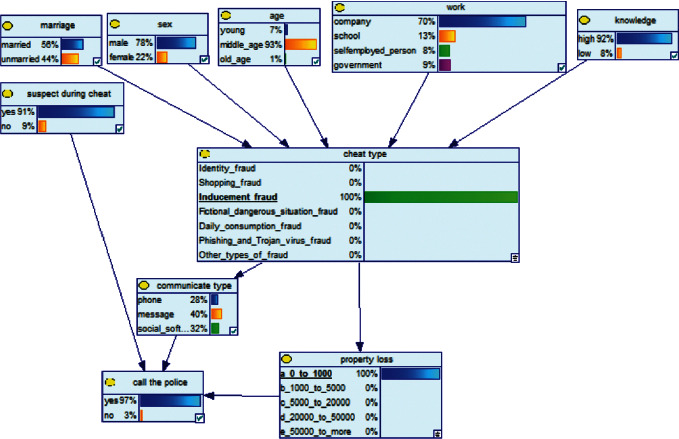
Scenario 4: Bayesian network probability distribution.

Combining the content of Figure 7, it can be seen that under the situation of Scenario 4, the probability of the victim being male is 78%, the probability of being middle-aged is 93%, the probability of being engaged in an occupation in the company is 70%, and the probability of having a high level of knowledge was 92%. According to the reverse reasoning of the two scenarios, middle-aged Kochi uncles who work in such companies are more susceptible to fraud. Therefore, when formulating preventive countermeasures in the future, public security decision-making departments should combine manual and technological means to focus on such groups, push targeted promotions, and publicize fraud cases that fit their identities, so that the publicized groups pay attention to antifraud work.

#### 3.4.2. Sensitivity Analysis

In order to determine the key parameters of the subnodes of telecom fraud results and quantitatively verify the Bayesian network model, a sensitivity analysis was carried out in this study. Sensitivity analysis refers to the sensitivity of small changes in input parameters to the impact of model performance [22]. In order to determine the parameters that have a significant impact on the final output results, and to take relevant antifraud measures such as prevention, publicity, and an interception in a timely manner, it is necessary to conduct a sensitivity analysis. The nodes of the Bayesian network are shown in Table 5.

**Figure 8:**
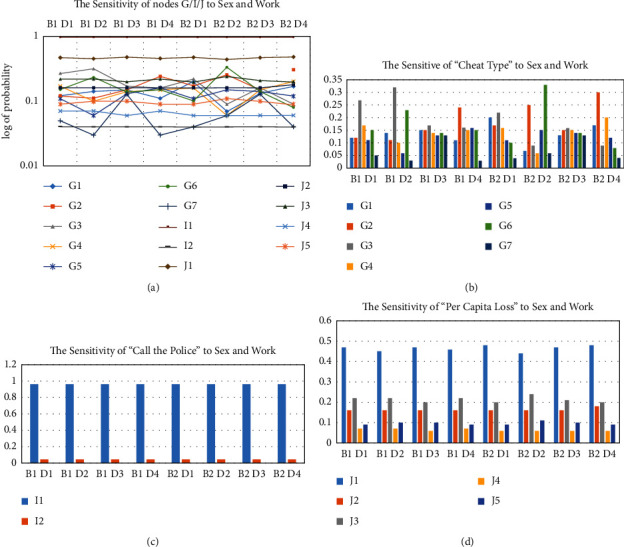
(a) The sensitivity of nodes G/I/J to sex and work. (b) The sensitivity of “Cheat Type” to sex and work. (c) The sensitivity of “Call the Police” to sex and work. (d) The sensitivity of “Per Capita Loss” to sex and work.

This article constantly explores the influence of people of different genders and positions on the type of deception, loss, and processing options (whether to report to the police) when they are defrauded because this will facilitate the public security organs to formulate targeted measures to publicize and prevent telecom fraud. We performed a sensitivity analysis in the set of eight types of cases (*B*_1_*D*_1_–*B*_2_*D*_4_), resulting in the probability results shown in Table 6. At the same time, we display the data results with a visual polyline of logarithmic values, as shown in Figure 8(a), in which it can be intuitively known that when the fraud type is *G*_7_, it changes the most with different cases, and whether *I*_1_ and *I*_2_ in the alarm it does not change with the change of the case, *I*_1_ maintains a high value, and *I*_2_ maintains a low value.

According to the probability distribution of fraud-type nodes obtained by the Bayesian network model, it can be found that gender and occupation have the least influence on “processing choice,” which is basically unchanged, and the influence of other nodes does not change much, and most of them are hovering between 1% and 5%. As shown in Figure 8(b), when the gender is male and the occupation is a government agency, the maximum probability of shopping fraud changes by 19%. At the same time, when the gender is female and the occupation is school, the maximum change in the probability of phishing and virus fraud reaches 19%. This reveals that certain types of fraud targets are targeted choices, which review the common knowledge of fraud but still need to be based on the Bayesian network model to dig out detailed potential relationships and take corresponding precautions.

When analyzing the sensitivity of the alarm node in the Bayesian network from Figure 8(c), we find that the value of the alarm attribute has not changed, the probability of selecting an alarm is 96%, and the probability of selecting no alarm is 4%, so the sensitivity of the node is very small, and there is basically no impact. The existence of this situation is also a good result of the recent antifraud propaganda of “calling the police in time when encountering fraud,” which has penetrated into the hearts of the masses, showing from the side that the public security organs are deeply trusted.

Next, focus on the sensitivity of property loss. In Figure 8(d), the property loss probability fluctuates around 5%, and the overall trend of change is not large. The most obvious changes are when the property loss is 0–1000 and 20000–50000. Figure 8-an and common sense in life show that the loss-generating people in these two situations approaching both ends covet a certain small gain, and the performance of this psychological behavior is related to gender and occupation to a certain extent, so it shows that the loss varies in different ways. Floatability under the case. At the same time, according to the results of sensitivity analysis, the public security organs should focus on key relationship groups to avoid large losses and protect people's property safety.

#### 3.4.3. Partial Verification

In order to quantitatively verify the proposed Bayesian network-based model, a partial verification method based on three axioms is also introduced in this study. The three axioms [23] are as follows:

1. If the prior probability of the parent node decreases/increases slightly, the posterior probability of the corresponding child node should also change accordingly.
2. Since the probability distribution of the parent node changes, it should have a consistent impact on the child nodes.
3. The magnitude of the total effect on the value of a combination of probability changes from *m* attributes should always be larger than from *m* − *n* (*n* ∈ *m*).

For example, considering the gender of the parent node of the fraud type, when the prior probability of the state “male” is set from 72% to 90%, the probability of shopping fraud drops from 16% to 15%, and the probability of property loss of 5000–20000 increases from 21% to 22%. Based on this change, when the prior probability that the parent node occupation is in the “school” state is set from 12% to 37%, the high probability of the parent node's occupation is set from 15% to 14%, and the probability of property loss of 5000–20000 is reduced from 21.6% to 21.8%. The process of adding each influence node satisfies the axioms and provides partial validation of the model. Perform a similar analysis on other nodes.

## 4. Discussion

The Bayesian network model based on victim telecom fraud proposed in this paper has a very good role in guiding, judging, and making decisions in the actual work of public security. One is to be able to make positive inferences from the attributes of the victims, to calculate the potential types of fraud and the amount of loss, to improve the ability of community fraud risk research and judgment, to add fraud research and judgment functions, and to improve the community risk research and judgment mechanism. Reverse reasoning, analyze the correlation between the type of case, the amount of fraud and other fraud results, and the victim's attributes, help guide the public security organs to discover the victim group, make auxiliary decision-making on fraud prevention measures and methods, and not only limit the antifraud countermeasures to experts Experience, using technology to improve accuracy and precision.

At the same time, the Bayesian network model can also use the change of external objective factors to affect the probability distribution of fraud result nodes to explore the effectiveness of external objective factors, dynamically evaluate the fraud situation, and adjust the combat strategy in time. For example, in terms of police crackdowns, when a certain type of case is found to have increased significantly in recent months, there will generally be special operations to crack down. After the arrest of one type of criminal gang, such crimes in the area will plummet. However, due to the cross-regional nature of telecom fraud and many criminal gangs from overseas, there will still be a large number of telecom fraud cases in this region. But a certain type may decline. In this case, a certain probability of a certain node will change, and at the same time, the characteristics of the deceived person in the objective conditions will also change. This requires us to perform a dynamic evaluation.

The Bayesian network cannot dynamically supplement knowledge, and at the same time, it cannot segment fine-grained nodes, and has the limitation of a single function. Therefore, the future development of this model can also be integrated with the knowledge graph. The same type of people may encounter different frauds to push similar cases. At the same time, it is necessary to push based on probability estimates, which will increase its accuracy, and reduce the push of spam information and targeted push, and can greatly improve the acceptability of the audience and increase the number of reading visits.

## 5. Conclusion

Identifying and assessing wire fraud types and losses is an important element of wire fraud prevention and decision making. In the research of this paper, we propose a Bayesian network model based on fraud victims to predict the types of fraud victims may encounter, possible fraud losses, etc. In order to effectively estimate the impact of the consequences of electrical fraud, this paper firstly conducts a literature review to identify the main consequence factors representing nodes in this Bayesian network model, and secondly establishes the causal relationship between nodes based on expert knowledge and published literature. Finally, use scenario analysis to predict the consequences of different scenarios, and identify influential factors through sensitivity analysis.

Due to the limited transmission of case data or information, such as telecom fraud cases, cases often occur due to the lack of attention of the audience. The Bayesian network-based telecom fraud victim consequence model provides a unique tool for public security organs and community government departments. The model can provide quantitative and qualitative information about different influencing factors of the consequences of fraud cases, and can highlight the most sensitive and vulnerable victim attribute nodes in fraud cases. At the same time, the developed BN-based telecom fraud victim consequences model can flexibly consider more factors, accommodate more information, and prioritize victim attributes set by relevant agencies. The effectiveness of the developed model directly depends on the information of the historical telecom fraud case data to generate the CPT of the Bayesian network structure. Since decision makers have different knowledge and experience, experts can be assigned weights or reputation scores to decision makers. For future research, the developed result model can be integrated with the knowledge graph to develop a telecom fraud risk early warning framework and realize a more comprehensive telecom fraud prevention system.

## Figures and Tables

**Table 1:** Comparison of fraud prevention work.

Previous work	Method	Limitation	Solution suggestion
Suspicious fraud detection based on the white box [2]	Use the set rules to judge the behavior data of people	Rules are fixed, inflexible, and cannot accommodate new forms of fraud	It is suggested to mind the fixed process factors of fraudulent behavior and determine the fraudulent behavior by exploring the interaction between factors.

Security system based on neural network classifier [5]	Using the neural network method to classify the characteristics of mobile phone operation behavior to determine whether there is dangerous behaviour	Relying on existing operational features, it is easy to be attacked by adversarial samples	It is suggested to use the Bayesian network method to realize the detection of security by exploring the interaction between the operational feature factors.

Recognition method based on GA-SVM [8]	Divide the call record data set into subsets using the k-mean method, and then use the subsets on the model as a training set and a cross-validation set	Single data, no process factors are taken into account	It is recommended to expand the variety of data types in the dataset

Fake account identification based on the Markov network [9]	By considering the group characteristics of telecommunication fraud, the Markov network is used to identify the transaction accounts, and the fraudulent accounts are excavated.	The scope of application is small, and the whole process of fraud prevention cannot be achieved	It is recommended to integrate this method into the whole process fraud prevention system

**Table 2:** Design of Bayesian network nodes for telecom fraud.

Nodes type	Nodes (BN variables)	States of Bayesian nodes
Portrait	(A) Sex	(1) Male, (2) female
	(B) Age	(1) Youth, (2) middle age, (3) old age
	(C) Marriage	(1) Married (2) unmarried
	(D) Work	(1) Company, (2) school, (3) selfemployed person, (4) government
	(E) Knowledge	(1) High, (2) low

Fraud process	(F) Cheat type	(1) Identity fraud, (2) shopping fraud, (3) inducement fraud, (4) fictional dangerous situation fraud, (5) daily consumption fraud, (6) phishing and Trojan virus fraud, (7) other types of cheat
	(G) Community type	(1) Phone, (2) message, (3) social software
	(H) Suspect during cheat	(1) Yes, (2) no
	(I) Call the police	(1) Yes, (2) no

Scam results	(J) Property loss	(1) 0–1000; (2) 1000–5000; (3) 5000–20000; (4) 20000–50000; (5) 50000+

**Table 3:** Estimated property loss of telecom fraud.

	*L *_1_	*L *_2_	*L *_3_	*L *_4_	*L *_5_
Value	0–1000	1000–5000	5000–20000	20000–50000	50000+
Probability	0.47	0.16	0.21	0.07	0.09

Max	9,8970 Yuan				
Average	1,4710 Yuan				
Min	8550 Yuan				

**Table 4:** Situational reasoning probability distribution table.

Attribute	State 1	State 2	State 3	State 4
Marriage	Married	Unmarried	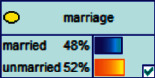	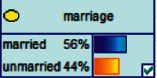
Sex	Male	Female	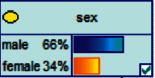	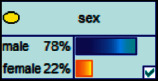 2
Age	Middle age	Old age	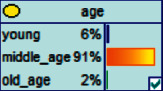	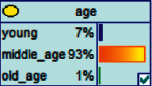
Work	Company	Selfemployed person	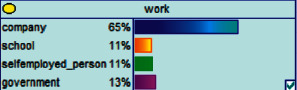	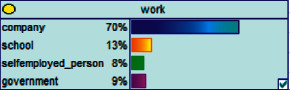 4
Knowledge	High	Low	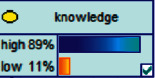	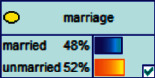 5
Suspect during cheat	Yes	No	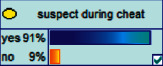 0	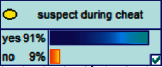 6
Cheat type	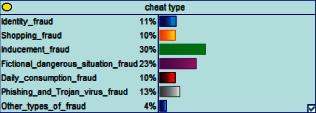	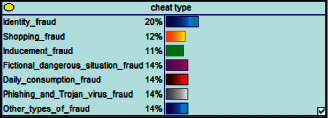	Identity fraud	Inducement fraud
Property loss	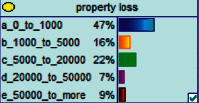	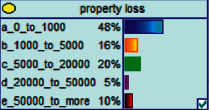	50000+	0–1000

**Table 5:** Bayesian network node table description.

Case	Bayesian nodes	Description
*A *_1_	(A) Marriage	Marriage = married
*A *_2_	(A) Marriage	Marriage = unmarried
*B *_1_	(B) Sex	Sex = male
*B *_2_	(B) Sex	Sex = female
*C *_1_	(C) Age	Age = young
*C *_2_	(C) Age	Age = middle age
*C *_3_	(C) Age	Age = old age
*D *_1_	(D) Work	Work = company
*D *_2_	(D) Work	Work = school
*D *_3_	(D) Work	Work = selfemployed person
*D *_4_	(D) Work	Work = government
*E *_1_	(E) Knowledge	Knowledge = high
*E *_2_	(E) Knowledge	Knowledge = low
*F *_1_	(F) Suspect during cheat	Suspect during cheat = yes
*F *_2_	(F) Suspect during cheat	Suspect during cheat = no
*G *_1_	(G) Cheat type	Cheat type = identity fraud
*G *_2_	(G) Cheat type	Cheat type = shopping fraud
*G *_3_	(G) Cheat type	Cheat type = inducement fraud
*G *_4_	(G) Cheat type	Cheat type = fictional dangerous situation fraud
*G *_5_	(G) Cheat type	Cheat type = daily consumption fraud
*G *_6_	(G) Cheat type	Cheat type = phishing and Trojan virus fraud
*G *_7_	(G) Cheat type	Cheat type = other types of fraud
*H *_1_	(H) Communicate type	Communicate type = phone
*H *_2_	(H) Communicate type	Communicate type = message
*H *_3_	(H) Communicate type	Communicate type = social software
*I *_1_	(I) Call the police	Call the police = yes
*I *_2_	(I) Call the police	Call the police = no
*J *_1_	(J) Property loss	Property loss = 0–1000
*J *_2_	(J) Property loss	Property loss = 1000–5000
*J *_3_	(J) Property loss	Property loss = 5000–20000
*J *_4_	(J) Property loss	Property loss = 20000–50000
*J *_5_	(J) Property loss	Property loss = 50000+

**Table 6:** Sensitivity analysis results under the characteristic node of Bayesian network.

Bayesian nodes	State of Bayesian nodes	Estimated probability							
		*B *_1_ * D *_1_	*B *_1_ * D *_2_	*B *_1_ * D *_3_	*B *_1_ * D *_4_	*B *_2_ * D *_1_	*B *_2_ * D *_2_	*B *_2_ * D *_3_	*B *_2_ * D *_4_
(G) Cheat type	*G *_1_	0.12	0.14	0.15	0.11	0.20	0.07	0.13	0.17
(G) Cheat type	*G *_2_	0.12	0.11	0.15	0.24	0.17	0.25	0.15	0.30
(G) Cheat type	*G *_3_	0.27	0.32	0.17	0.16	0.22	0.09	0.16	0.09
(G) Cheat type	*G *_4_	0.17	0.10	0.14	0.15	0.16	0.06	0.15	0.20
(G) Cheat type	*G *_5_	0.11	0.06	0.13	0.16	0.11	0.15	0.14	0.12
(G) Cheat type	*G *_6_	0.15	0.23	0.14	0.15	0.10	0.33	0.14	0.08
(G) Cheat type	*G *_7_	0.05	0.03	0.13	0.03	0.04	0.06	0.13	0.04
(I) Call the police	*I *_1_	0.96	0.96	0.96	0.96	0.96	0.96	0.96	0.96
(I) Call the police	*I *_2_	0.04	0.04	0.04	0.04	0.04	0.04	0.04	0.04
(J) Property loss	*J *_1_	0.47	0.45	0.48	0.46	0.48	0.44	0.47	0.48
(J) Property loss	*J *_2_	0.16	0.16	0.16	0.16	0.16	0.16	0.16	0.18
(J) Property loss	*J *_3_	0.22	0.22	0.20	0.22	0.20	0.24	0.21	0.20
(J) Property loss	*J *_4_	0.07	0.07	0.06	0.07	0.06	0.06	0.06	0.06
(J) Property loss	*J *_5_	0.09	0.10	0.10	0.09	0.09	0.11	0.10	0.09

## Data Availability

The data used to support the findings of this study are available from the corresponding author upon request.
